# Diclofenac-induced thrombotic thrombocytopenic purpura with concomitant complement dysregulation: a case report and review of the literature

**DOI:** 10.1186/s13256-019-2097-5

**Published:** 2019-06-23

**Authors:** Jose Perez Lara, Yaneidy Santana, Maneesh Gaddam, Asghar Ali, Sandeep Malik, Misbahuddin Khaja

**Affiliations:** 1 Department of Medicine, Bronx Care Health System, Affiliated with Icahn School of Medicine at Mount Sinai, 1650 Grand Concourse, Bronx, NY 10457 USA; 2 Division of Pulmonary and Critical Care Medicine, Bronx Care Health System, Affiliated with Icahn School of Medicine at Mount Sinai, 1650 Grand Concourse, Bronx, NY 10457 USA; 3 Division of Hematology and Oncology, Bronx Care Health System, Affiliated with Icahn School of Medicine at Mount Sinai, 1650 Grand Concourse, Bronx, NY 10457 USA

**Keywords:** Thrombotic thrombocytopenic purpura, Hemolytic uremic syndrome, Non-steroidal anti-inflammatory drug, Thrombotic microangiopathies

## Abstract

**Background:**

Thrombotic thrombocytopenic purpura and hemolytic uremic syndrome are two forms of thrombotic microangiopathies. They are characterized by severe thrombocytopenia, microangiopathic hemolysis, and thrombosis, leading to a systemic inflammatory response and organ failure. Plasmapheresis is used to treat thrombotic microangiopathies. A different entity known as atypical hemolytic uremic syndrome has garnered more clinical recognition because reported cases have described that it does not respond to standard plasmapheresis. Diclofenac potassium is a non-steroidal anti-inflammatory drug that is used to treat pain.

**Case report:**

A 35-year-old Hispanic man presented to our emergency department with complaints of generalized malaise, fever, and an evanescent skin rash. During admission, he reported the use of diclofenac potassium for back pain on a daily basis for 1 week. He was noted to have peripheral eosinophilia, so he was admitted for suspected drug reaction involving eosinophilia and systemic symptoms. His initial laboratory work-up showed microangiopathic hemolytic anemia and thrombocytopenia. He also experienced a seizure, encephalopathy, and had a PLASMIC score of 7, thus raising concerns for thrombotic thrombocytopenic purpura. He underwent emergent plasmapheresis, which improved his clinical condition. The diagnosis was confirmed by assessing the levels of disintegrin and metalloproteinase with thrombospondin type 1 motif, member 13, which was less than 3%. In addition, his skin biopsy was positive for patchy complement deposition, demonstrating complement dysregulation.

**Conclusion:**

Thrombotic thrombocytopenic purpura is a rare condition that can be acquired. Our case is rare because it represents the first report of diclofenac potassium-induced thrombotic thrombocytopenic purpura with subjacent complement activation and dysregulation. Early recognition and aggressive management led to a favorable outcome.

## Introduction

Thrombotic thrombocytopenic purpura (TTP) is a medical emergency and a form of thrombotic microangiopathy (TMA), in which the activity of the von Willebrand factor-cleaving protease, a disintegrin and metalloproteinase with thrombospondin type 1 motif, member 13 (ADAMTS13), is reduced. It can be either hereditary or acquired [1].

Only 5% of cases will involve the pentad of criteria for the diagnosis of TTP: fever, microangiopathic hemolytic anemia, thrombocytopenia, renal involvement, and neurologic involvement. The acquired form of TTP is due to an autoantibody inhibitor, and the hereditary form is due to mutations in ADAMTS13.

Hemolytic uremic syndrome (HUS) is another form of TMA [2]. Typical HUS has manifestations of microangiopathic hemolytic anemia, thrombocytopenia, renal failure, diarrhea, and abdominal pain. The gastrointestinal symptoms are secondary to a Shiga-like toxin from *Escherichia coli*. Atypical HUS (aHUS) is secondary to mutations in the complement pathway C3–C4 [3].

Differentiating between aHUS and TTP, particularly acquired TTP, presents a clinical challenge, as both types share similar initial findings of thrombocytopenia, microangiopathic hemolytic anemia, and diffuse organ damage. However, assessing the clinical presentation can provide clues on renal function, which differs between aHUS and TTP. Renal injury is more frequent in aHUS, whereas neurological symptoms are more common in TTP. Low serum levels of ADAMTS13 or the demonstration of complement mutation can be used to give a definitive diagnosis of TTP [4].

Diclofenac potassium is a non-steroidal anti-inflammatory drug (NSAID) that is used for the treatment of inflammation and pain. Common side effects of NSAIDs include gastrointestinal bleeding, ulceration, perforation, anemia, thrombocytopenia, and an abnormal liver function test. NSAIDs also increase the risk of myocardial infarction and stroke [5].

We present a rare case of diclofenac potassium-induced TTP (acquired hemolytic microangiopathy anemia) with subjacent complement activation and dysregulation as shown in dermatopathology from a skin biopsy. We describe a patient in whom plasmapheresis was not readily available and immunomodulators, such as eculizumab, could potentially be used as a bridge therapy for such cases.

## Case presentation

**Fig. 1:**
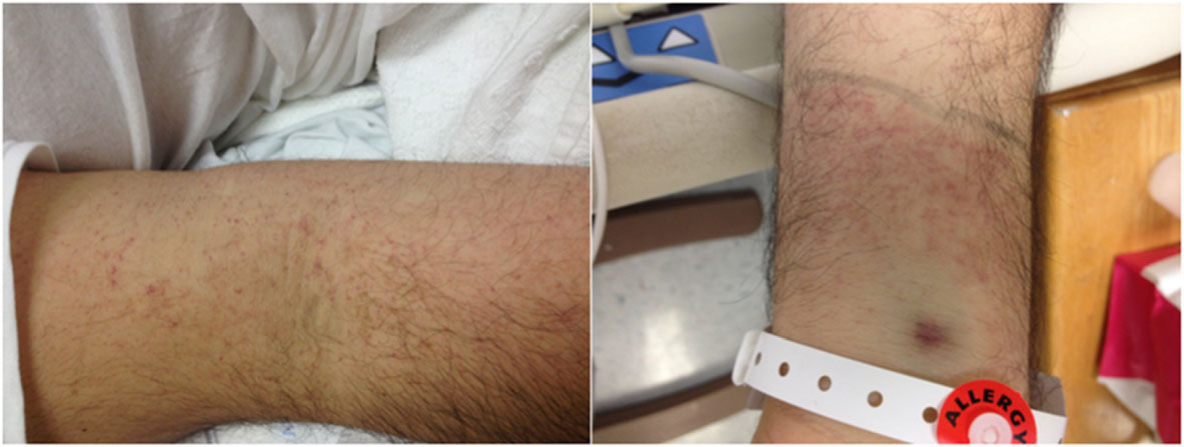
Petechial skin rash

A 35-year-old Hispanic man presented to our emergency department and reported 1 week of generalized malaise, abdominal pain, fatigue, subjective fever, sore throat, joint pain, watery diarrhea, intolerance to oral intake, and petechial skin rash (Fig. 1). The skin rash was described as blisters, which progressed to erythematous macules that were non-confluent and were associated with burning and itching. The rash started on his palms and soles and then subsequently spread centripetally to his arms, chest, and trunk. Diarrhea occurred at a frequency of 2–3 episodes a day but had resolved by the time of evaluation. His past medical history was only significant for chronic back pain, for which he had been taking 100 mg of diclofenac potassium twice daily for 1 week before admission. Prior to admission, he was not taking any medications due to lack of a past medical history. The only medication taken was diclofenac potassium, as mentioned above. He denied the use of any other medication, tobacco smoking, alcohol intake, or drug use. His family history and psychosocial history were unremarkable. He denied any prior surgical intervention. He reported self-employment as an independent taxi driver.

At initial assessment on admission the physical examination was remarkable for a blood pressure of 132 systolic and 77 diastolic mmHg, a temperature of 39.6 °C (103.2 °F), pulse of 132, and respiratory rate of 18. He was well developed, well groomed, with skin remarkable for non-confluent, non-blanching erythematous macules. He had an atraumatic head and his eyes had reactive pupils that were symmetric with clear conjunctiva. He had a supple neck with no signs of jugular vein distention (JVD) or thyromegaly. His thorax was symmetric with non-labored respirations, lungs were clear to auscultation bilaterally, his heart rate was regular with regular rhythm, pulses were palpable and normal in his bilateral upper and lower extremities; no rub or murmur was noted. His abdomen was soft and non-tender with no distention and no palpable masses; bowel sounds were present. He was alert and oriented, and followed commands; cranial nerves 2–12 were preserved, deep tendon reflexes were intact, strength was 5/5 bilaterally in both his upper and lower extremities.

The laboratory results were remarkable for anemia, thrombocytopenia, elevated C-reactive protein (CRP) 21.9 mg/L (reference value ≤ 5 mg/L), and mild transaminitis, with no deranged bilirubin or alkaline phosphatases. No acute findings were reported from chest X-ray.

He was initially diagnosed as having acute viral gastroenteritis and was managed with supportive therapy. Due to a persistent fever, he was transferred to the intensive care unit (ICU) and started on broad-spectrum antibiotics and antiviral therapy, which included vancomycin, aztreonam, azithromycin, and oseltamivir. Because his blood cultures and urine culture were negative, vancomycin was discontinued.

**Fig. 2:**
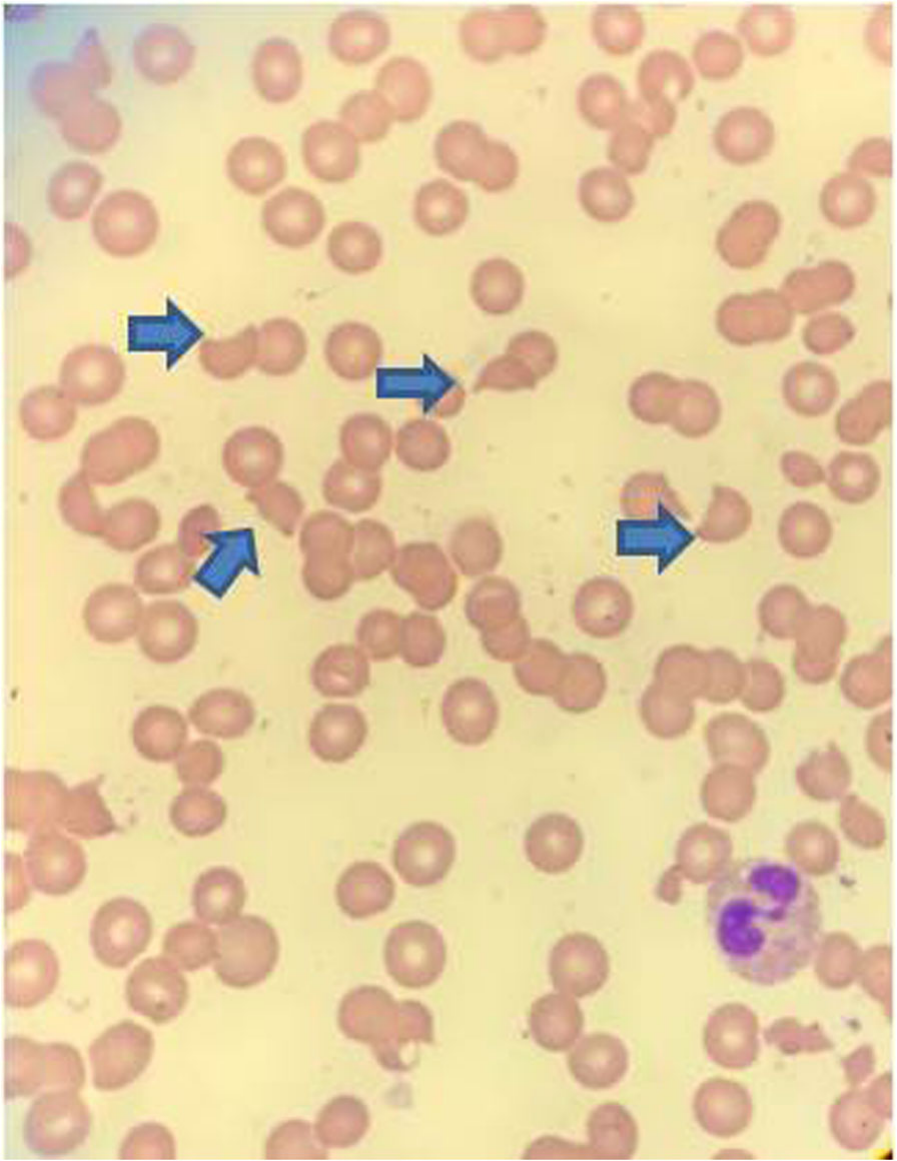
Peripheral smear with schistocytes (arrows)

**Fig. 3:**
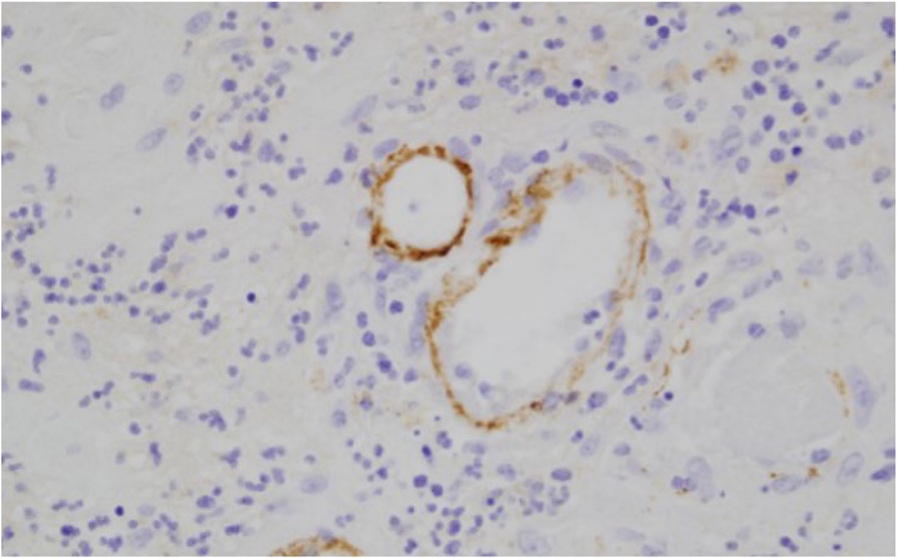
Immunofluorescent stain of skin biopsy showing patchy granular deposition of C5b-9 along superficial dermal vessels

His febrile episodes persisted and he had repeated negative blood and urine cultures, negative vasculitis, and unremarkable autoimmune work-ups. Subsequently, he developed acute encephalopathy and experienced a seizure. He was then intubated for airway protection and placed on mechanical ventilation. A lumbar puncture was deferred, given the presence of profound thrombocytopenia and the risk of bleeding. Computed tomography of his head reported no acute signs of intracranial hemorrhage or infarction. Other remarkable findings were a platelet count of 10 k/μl, hemoglobin of 6.2 g/dl, hematocrit of 18.2%, reticulocyte count of 10%, plasma prothrombin time (PT) > 11.3 seconds (reference range, 9–12 seconds), activated partial thromboplastin time (PTT) > 27.1 seconds (reference range, 26.1–33.8 seconds), serum creatinine 0.8 mg/dl (reference range, 0.5–1.5 mg/dl) alanine aminotransferase serum of 55 unit/L (reference range, 5–40 unit/L), aspartate transaminase serum of 35 unit/L (reference range, 9–48 unit/L), total bilirubin serum of 1.4 mg/dL (reference range, 0.2–1.2 mg/dL), direct serum bilirubin of 0.6 mg/dL (reference range, 0.0–0.3 mg/dL), lactate dehydrogenase (LDH) serum of 1172 unit/L (reference range, 100–190 unit/L), haptoglobin of 0 mg/dL (reference range, 30–200 mg/dL), a peripheral smear with schistocytes (Fig. 2), total hemolytic complement assay less than 14 (reference range, 31–60 U/mL), and a positive direct Coombs test (Table 1). All viral serologic tests were negative (Table 2). Given these test results and a significantly worsening neurological status, high doses of intravenously administered steroids were initiated, along with intravenously administered gamma immunoglobulin. This was indicated for suspected immune-mediated hemolysis versus an acute vasculitis process. A skin biopsy was also performed, and a formalin-fixed skin biopsy showed patchy granular deposition of C5b-9 along superficial dermal vessels, dermal epidermal junction (Fig. 3).

**Table 1:** Laboratory findings during admission

Day of admission	0	3	7	9	11	12	Time of discharge	Clinic follow-up in 2 weeks
Hemoglobin (g/dl)	10.5	8.7	6.9	6.2	10.3	11.7	12.9	13.9
Platelets (k/μl)	10	10	21	67	134	160	369	255
Haptoglobin (mg/dl)	5	4	1	0	0	45	63	188
Indirect bilirubin (mg/dl)	0.3	1.3	0.7	0.7	0.8	0.8	0.6	0.4
Lactate dehydrogenase (unit/L)	524	538	984	692	572	259	208	217
Reticulocyte count (%)	6	7	10	11	15.8	15.3	10	4.8
ADAMTS13 (%)	–	–	–	< 3	–	–	117	126
PT (9.5–12 seconds)	11.3	13.5	13.4	12.9	10.8	–	10.8	–
INR (0.0–2.0)	1.1	1.1	1.1	1.1	0.9	–	0.9	–
PTT (26.1–33.8 seconds)	27.1	24.9	27.3	23.5	22.8	–	24.8	–
Fibrinogen (185–450 mg/dL)	–	380	–	405	–	–	–	–

*ADAMTS13* disintegrin and metalloproteinase with a thrombospondin type 1 motif, member 13, *INR* international normalized ratio, *PT* prothrombin time, *PTT* partial thromboplastin time

**Table 2:** Virologic test results

Test	Results
Parvovirus B19 IgM	0.4 (< 0.9)
Rubella IgM	< 20
EBV-VCA IgM	43
CMV IgM	< 30
Influenza	Negative
Dengue IgM	< 1.1 (1.65)
Chikungunya	Negative
HIV	Negative
RSV A/B	Not detected
Human parainfluenza virus	Not detected
Human metapneumovirus	Not detected
Rhino virus	Not detected
Enterovirus	Not detected
Coxsackie virus	Not detected

*CMV* cytomegalovirus, *EBV-VCA* Epstein–Barr virus viral capsid antigen, *HIV* human immunodeficiency virus, *RSV* respiratory syncytial virus, *IgM* Immunoglobulin M

The findings of microangiopathic hemolytic anemia, thrombocytopenia, and neurological derangement were enough to suspect a diagnosis of TTP. Thus, serum ADAMTS13 activity and inhibitor assays were conducted. Serum ADAMTS13 activity was < 3% (reference range, 68–163%), and the serum inhibitor level was 1.5 (normal range, < 0.5). Specific identification of the inhibitor could not be obtained. He was then empirically treated by transfusing fresh frozen plasma and he underwent plasmapheresis sessions.

He received five sessions of plasmapheresis, which improved his clinical status and allowed for successful liberation from mechanical ventilation. Antibiotics were de-escalated based on culture reports, and intravenous steroid dosages were tapered down. Laboratory assessments showed increases in levels of platelets, haptoglobin, LDH, and hemoglobin. Levels of important compounds throughout the course of treatment are presented in Table 1. He was subsequently discharged from the ICU and hospital. The post-plasmapheresis level of serum ADAMTS13 activity was 126%, thus confirming the diagnosis of acquired TTP.

Further follow-ups were done in our hematology clinic at 3 months and 6 months following hospital discharge. As mentioned in this case report, he was found to be asymptomatic, including a lack of rash and no further neurological symptoms. In addition, all laboratory tests were reported to be within normal limits, including serum haptoglobin, LDH, hemoglobin, and platelets, as were renal function tests, liver function tests, and serum ADAMTS 13 activity. No further intervention was recommended from a hematology point of view and our patient returned to work without further complications.

Our patient gave us informed consent to publish this case, including images.

## Discussion

In summary, we presented a 35-year-old, Hispanic man whose self-administration of diclofenac potassium triggered an episode of secondary acquired TTP which presented initially as a skin rash. Only a few cases have been reported where NSAID use has been linked to acquired TTP and none have been associated specifically to exposure of diclofenac potassium. Furthermore, skin rash is an uncommon presentation for TTP and is explained by complement dysregulation and deposition in the dermis.

TTP can result from ADAMTS13 deficiency and can either be hereditary or acquired. It was first described by Moschcowitz in 1924; HUS was first described by Gasser in 1955 [6].

In the acquired form of TTP, several risk factors can trigger the formation of antibodies against ADAMTS13 or damage the endothelial cells to liberate a large amount of ultra-large von Willebrand factor. Some of the identifiable risk factors include immunosuppressive therapy, human immunodeficiency virus (HIV) infections, malignancy, pancreatitis, post-surgical state, post-partum state, and post-pneumococcal infection state. Standard drugs that can cause TTP include antibiotics, oral contraceptive pills, extended-release opioids, valacyclovir, as well as chemotherapeutic agents such as mitomycin C, alkylating agents, and immunomodulators [3, 7]. In rare cases, NSAIDs may also cause TTP.

The incidence of acquired TTP is 4–10 cases per 1 million adults per year, with the median age being 41 years. Female sex and black race are increased risk factors for TTP [8]. Common presenting symptoms include nausea, vomiting, abdominal pain, dizziness, bruising, and weakness. Some of the other features include dyspnea, petechiae, bleeding, and fatigue [9]. Neurological findings may consist of transient ischemic episodes, numbness, weakness, dysarthria, seizure, and coma [10].

If TTP is suspected, the following laboratory tests should be performed: a complete blood count including platelet count, peripheral blood smear, serum chemistry, creatinine, serum LDH, serum bilirubin, haptoglobin, a coagulation profile including PT, activated PTT, fibrinogen, D-dimer levels, Coombs test, and ADAMTS13 activity and inhibitor levels.

In this case, our patient presented with non-specific symptoms and a viral-like syndrome with a purpuric skin rash. However, the development of acute microangiopathic hemolytic anemia (that is, low haptoglobin, elevated LDH, and schistocytes in the peripheral smear), acute encephalopathy, thrombocytopenia, and low serum ADAMTS 13 activity were enough evidence to suspect TTP and to start plasmapheresis. HUS and aHUS were also part of the differential diagnosis, although the lack of firm evidence of significant renal impairment favored TTP diagnosis. Pre-treatment serum levels of ADAMTS13 were assessed by the laboratory, and a skin biopsy was also performed as part of the work-up for aHUS to assess complement deposition in dermal vessels and the dermo–epidermal junction [11].

ADAMTS13 is a von Willebrand factor-cleaving protease. Levels less than 5% suggest 100% sensitivity and specificity for the diagnosis of TTP. In the absence of ADAMTS13, the large von Willebrand factor multimers attach to platelets, leading to the formation of platelet thrombi. The acquired form of TTP is suggested by the presence of the ADAMTS13 inhibitor [12].

In addition, the PLASMIC score system can predict levels of ADAMTS13 activity. It is composed of the following criteria: a platelet count < 30,000/μL, evidence of hemolysis (reticulocyte count > 2.5%, elevated indirect bilirubin > 2 mg/dL, undetectable to low haptoglobin levels), mean corpuscular volume < 90 fl, international normalized ratio < 1.5, and creatinine < 2 mg/dl with no active cancer or organ/stem cell transplant. This scoring system gives one point to each of the above met criteria. A low score of 0–4 suggests an ADAMTS13 activity of 10% or greater, and a score of 6–7 predicts ADAMTS13 activity to be less than 10%, with a sensitivity of 91% [13]. Our patient’s PLASMIC score was 7.

Furthermore, in laboratory we found evidence of complement consumption given absolute decrease in total hemolytic complement suggesting inadequate complement activation which can suggest aHUS as a differential diagnosis. Our patient’s skin biopsy was positive, due to the presence of patchy C5b-9 complement deposition on the dermal–epidermal junction. However, intense diffuse deposition was not reported. Thus, the diagnosis of aHUS was not supported. It was determined that he had a diagnosis of TTP with combined complement dysregulation. This was further supported by low serum levels of ADAMTS13 prior to plasmapheresis and an improvement in clinical status, hemolytic anemia parameters, and thrombocytopenia shortly after plasma exchange therapy. The effectiveness of plasmapheresis officially ruled out aHUS as a possible diagnosis, as aHUS does not respond to plasmapheresis treatment. Other pertinent results related to our case were the negative findings for several viral and parasitic infections that were considered in the differential diagnosis, these included: dengue virus fever, HIV testing, malaria smear, strongyloidiasis serum antigen, parvovirus B19 Immunoglobulin M (IgM), and Lyme disease (Table 2).

Of importance to mention, TTP has been associated with complement activation and consumption, findings that have been described since 1977. Although the exact mechanism has not yet been fully elucidated, it has been proposed to be via activation of alternative pathway through C3b and C5b-9 deposition in platelets or by secretion of C3a. In our case, we evidenced this complement dysregulation by the C5b-C9 deposition in dermo–epidermal junction of the skin biopsy. This is of importance because a patient with significant evidence of complement consumption may benefit from immunotherapy, such as eculizumab, as a bridging therapy along with steroids until plasmapheresis can be initiated [11–14].

Another atypical feature of this patient presentation was that after a thorough interview with the patient and family members, along with an extensive rheumatologic work-up, no significant trigger for the development of TTP was identified. The only positive finding was the use of diclofenac potassium for almost 1 week as a self-treatment for back pain.

Diclofenac potassium is a form of NSAID that relieves pain. The most common adverse effects of NSAIDs include gastrointestinal symptoms and renal failure. To the best of our knowledge, some rare adverse effects of NSAIDs include skin reaction, erythema multiforme, fixed drug eruption, urticaria, blood dyscrasias, acute hepatocellular damage, pneumonitis, and neurological effects (for example, dizziness, nausea, aseptic meningitis, and headache) [15].

Only a few cases have reported NSAID-triggered TTP; these cases specifically involved ibuprofen. In 1974, a 55-year-old Italian woman died after developing TTP that was triggered by ingesting 900 mg ibuprofen. An autopsy confirmed generalized microscopic thrombosis, along with punctiform hemorrhage. She was unsuccessfully treated with peritoneal dialysis [16]. Of note, plasmapheresis was not a treatment option at that time. Oregel *et al*. reported a case of a 21-year-old man who attempted suicide by ingesting ibuprofen and subsequently developed TTP, which responded favorably to plasma exchange therapy [7]. Benmoussa *et al*. also reported a case of a 37-year-old African American man who attempted suicide by ingesting 30 pills of ibuprofen (400 mg), which induced TTP [17]. Timely identification and proper treatment resulted in a favorable outcome [17].

We attribute diclofenac potassium as the cause of TTP because a list of common differentials was excluded. Disseminated intravascular coagulation (DIC) was excluded as fibrinogen, D-dimer levels, PT, and PTT were normal. In terms of sepsis, our patient was treated empirically with broad-spectrum antibiotics which were subsequently de-escalated after blood cultures, urine culture, stool culture, and serologies for strongyloidiasis returned negative. Fortunately, our patient was also not in shock. Although no source of infections was located in several surveillance cultures, it is important to recognize that our patient was in fact in a state of systemic inflammatory response syndrome (SIRS) which resolved after treatment for TTP. Given this patient is Hispanic and from the Dominican Republic, we tested him for most common causes of hemorrhagic fever, such as chikungunya virus and dengue virus. However, these tests returned with negative laboratory results. Also, he was tested for *Leptospira* and parvovirus which were also negative. Other etiologies such as *Rickettsia*, hantavirus, and other viral hemorrhagic fever causes (like yellow fever) were not tested given no recent travel and no exposure to mosquitoes, rodents, or other epidemiologic factor in our patient. He also did not have a high fever on presentation.

A false positive direct Coombs test has been reported with drugs like penicillin, cephalosporins, methyldopa, intravenous immunoglobulin (IVIG), HIV, malaria and conditions like lupus. A positive Coombs test may suggest immune hemolysis but other causes should be excluded as in our case [18–21].

In our case several of the secondary causes of a positive Coombs test were ruled out such as HIV and malaria. During the initial management of our patient in the ICU admission, he received 50 g of IVIG as empiric treatment for idiopathic thrombocytopenia, which was the incorrect initial diagnosis. In our case, we explained that the result of the Coombs test was likely a false positive given evidence of normal serum levels of IgA, IgM, and IgE (IgG was not considered given he had received IVIG), and the lack of evidence for autoimmune hemolysis, lack of exposure to penicillin or cephalosporins, and absence of autoimmune conditions such as lupus. A false positive Coombs test has been found incidentally with yet unknown significance, which raises the need for more research to reveal the pathophysiology underlying TTP.

To the best of our knowledge, our patient represents the first case of diclofenac potassium-induced TTP. It is especially important to remember this association, as patients are widely exposed to NSAIDs and can use them indiscriminately for pain control.

## Conclusion

Physicians and patients should be reminded that all medications, as benign as they might appear, can potentially trigger serious events, such as TTP, in a susceptible population. Lethal consequences may occur, if treatment is not initiated in a timely manner. TTP is a rare condition that can be acquired. Three cases of ibuprofen-induced TTP have been reported. This case is rare, as it represents the first report of diclofenac potassium-induced TTP in a patient with subjacent complement dysfunction, as illustrated by skin biopsy. Early recognition and aggressive management allowed for a favorable outcome.

## Abbreviations

ADAMTS 13: Disintegrin and metalloproteinase with a thrombospondin type 1 motif, member 13
aHUS: Atypical hemolytic uremic syndrome
HIV: Human immunodeficiency virus
HUS: Hemolytic uremic syndrome
ICU: Intensive care unit
IVIG: Intravenous immunoglobulin
LDH: Lactate dehydrogenase
NSAID: Non-steroidal anti-inflammatory drug
PT: Prothrombin time
PTT: Partial thromboplastin time
TMA: Thrombotic microangiopathies
TTP: Thrombotic thrombocytopenic purpura
